# News and Coming Events

**Published:** 1908-04-11

**Authors:** 


### NEWS AND COMING EVENTS.

The Hon. W. F. D. Smith, M.P., has been elected chairman of the committee of management of King's College Hospital.

The Queen and the Empress Marie on April 2 inspected the Alexandra Hospital for Children with Hip Disease, Queen Square, Bloomsbury.

The first International Congress on "First Aid and Life Saving" is to be held at Frankfort-on-Maine during the week beginning June 7, 1908.

The annual debate of the Hunterian Society was held at the London Institution on April 8, when Dr. Frederick Taylor opened a discussion on "Rheumatism."

Mr. W. C. Bridgeman, M.P., treasurer of the Hospital for Invalid Gentlewomen, has received on behalf of the hospital an anonymous donation of £1,000 to the building fund.

The annual meeting of the Hospital Saturday Fund will be held at the Mansion House to-day (Saturday, April 11). The Lord Mayor, President of the Fund, will take the chair at 4 p.m.

A home for lepers will shortly be built in St. Croix, one of the Danish colonies in the West Indies. The Order of Oddfellows in Copenhagen has collected the necessary funds, and it appears that the home is very badly wanted.

The Prince of Wales, as Chancellor of the University of Wales, has nominated the Senior Deputy-Chancellor, Sir Isambard Owen, to represent the University on the Court of Governors of the University of Sheffield.

The Great Western Railway Company has sent a donation of £250 towards the funds which are being raised on behalf of St. Mary's Hospital, Paddington, by the "Clarence Wing Fête," which is to be held at the end of May.

After the annual meeting of the Society for the Study of Inebriety, which will be held in the Rooms of the Medical Society of London, 11 Chandos Street, Cavendish Square, W., on Tuesday, April 14, at 4 p.m., a discussion on "Reasons for Drinking" will be opened by Mr. Stanley B. Atkinson, M.A., M.B., B.Sc., J.P., barrister-at-law.

At a court of committees of Guy's Hospital, held on April 3, the following gentlemen were elected as honorary anæsthetists to the institution—namely, Mr. H. M. Page, F.R.C.S., anæsthetist to the West London Hospital and the Belgrave Hospital for Children; Dr. F. E. Shipway, M.A., M.D., Cantab., anæsthetist to the Victoria Hospital for Children, Chelsea.

For the work in the out-patients' department at Kentish Town the following appointments have been made by the Council of the Hampstead General Hospital, on the recommendation of the Medical Committee :—Dr. C. O. Hawthorne, M.R.C.P., Dr. F. W. Price, M.R.C.P., Mr. J. W. Thomson Walker, F.R.C.S., Mr. W. Fedde Fedden, M.S., F.R.C.S.

In addition to these appointments, it it intended shortly to fill posts in the following special departments :—Gynæcology; eye; skin; and nose, throat, and ear.

At the annual meeting of the British Lying-in Hospital, Endell Street, the Chairman, Mr. Charles E. Farmer, drew attention to the fact that the hospital now possesses a resident medical officer. This appointment, which is for a term of four months, considerably increases the utility of the hospital, and enables cases of greater difficulty to be admitted and major operations to be performed.

Attention has lately been drawn to the fact that Mr. Ernest Harnack, head of the $x$-ray department of the London Hospital, and one of the earlier investigators in this branch of medical science, has undergone four operations upon his hands as the result of $x$-ray dermatitis followed by malignant disease, and is now disabled. An appeal is being made for a testimonial to Mr. Harnack.

Owing to the very large number of urgent cases applying for relief the board of management of the Royal Hospital for Incurables, Putney Heath, have decided to elect 35 candidates at their next half-yearly election on May 29. The usual number of candidates elected has in the past been 25. The Royal Hospital for Incurables spends £35,000 a year in caring for those who are beyond medical and surgical skill.

A meeting of the Provisional Council of the National Union of Public Health Authorities was held on March 26 at the Grand Hotel, London, for the purpose of considering the rules of the Association and the transaction of other business. The draft rules prepared by the Chairman and the Hon. Secretary, after being carefully considered, and in a few respects amended, were adopted and ordered to be submitted to a Conference of the Union to be held on May 28 at Caxton Hall, Westminster.

On April 7, in the King's Bench Division, before the Lord Chief Justice, Mr. Justice Ridley, and Mr. Justice Darling, an appeal was heard against the decision of the Justices of Kingston-upon-Thames in the recent proceedings taken by the Royal College of Veterinary Surgeons against Matthew Collinson, under the Veterinary Surgeons Act of 1881. The respondent, although he was not a registered veterinary surgeon, had exhibited outside his house a board with these words upon it : "M. Collinson, canine specialist; dogs and cats treated for all diseases," and the Justices on February 1 acquitted him of infringement of the Act. Mr. Morton Smith appeared for the appellants. The appeal was allowed, and the case remitted to the magistrates.

The first block of the new King's College Hospital, at Denmark Hill, will comprise casualty, bathing and electrical, out-patients and dispensary departments, and the cost is £65,000. It is expected that the building will be completed within eighteen months. The plans for the administrative block, which is to be the next taken in hand, are in a very advanced state, and it is hoped that before the first portion is finished considerable progress will have been made with this section. After that will come the building of the ward blocks. In the first instance it is proposed to provide for some 350 beds, though the complete scheme makes provision for 600 beds and a medical school. Of the £300,000 required for the carrying out of the scheme up to the point at which 350 beds will be available £193,000 has been realised.

The King has sent a letter from Biarritz to Mrs. Hall Edwards in reply to a letter of thanks which she wrote in acknowledgment of the pension granted to her husband from the Civil List. His Majesty expressed his pleasure at conferring the pension upon Dr. Hall Edwards in recognition of his self-sacrificing services towards the advancement of science and the benefit of humanity.

H.R.H. The Duchess of Albany presided at a meeting of the Jubilee Committee of the National Hospital for the Paralysed and Epileptic (Albany Memorial), held at the Institution, Queen Square, Bloomsbury, on March 31. It is hoped by the efforts of the Committee to raise a sum of £50,000 to celebrate the jubilee of the hospital, which takes place next year. A portion of the money will be expended in effecting much-needed alterations and improvements, and the Committee hope to have a sufficient surplus for investment as a reserve fund for future contingencies. Various suggestions were made as to the best methods that should be adopted for the purpose of raising the amount required. Already £1,100 has been received or promised. The Duchess of Albany expressed her deep interest in the object of the Committee.

A meeting convened by the Lord Mayor was held on April 7 in the Manchester Town Hall to appeal for funds for the new Royal Infirmary, which is being erected in the neighbourhood of Manchester University. For the site of the existing infirmary, which has been in the middle of the city for 156 years, the corporation have agreed to pay £400,000, and the new buildings will cost £500,000. In the new hospital, which is designed to accommodate 600 beds, 500 will be at once provided, a number 208 larger than in the old building. In addition to the need of £100,000 for the building fund, an additional income of £12,000 a year will be required. The existing income, which just balances expenditure, is £22,500, and the estimated annual expenditure in the new building is £34,500 a year. At the meeting subscriptions amounting to £24,500 were announced.

Dr. Samuel Rideal gave on April 2, at the London School of Tropical Medicine, a demonstration of the Rideal-Walker test for determining the efficiency of disinfectants. The test was invented by Dr. Rideal in collaboration with Mr. Ainslie Walker in order to determine the relative values of germicides. Dr. Rideal used the typhoid bacillus for the purposes of the demonstration. The carbolic-acid co-efficient of each disinfectant under investigation was determined against a standard strength of carbolic. The test has been adopted by the War Office, the Admiralty, and other Government departments, as well as by municipal and other public authorities in Great Britain and the Colonies. Professor Hewlett, in the course of a discussion which followed, spoke of the dangers to the public of the sale of inefficient substances under the name of disinfectants, and urged upon the company the need for legislative control.

